# Wound complication after modified Ravitch for pectus excavatum: A case of conservative treatment enhanced by pectoralis muscle transposition

**DOI:** 10.1016/j.ijscr.2019.12.023

**Published:** 2019-12-26

**Authors:** Beatrice Aramini, Uliano Morandi, Giorgio De Santis, Lucio Brugioni, Alessandro Stefani, Ciro Ruggiero, Alessio Baccarani

**Affiliations:** a Division of Thoracic Surgery, Department of Medical and Surgical Sciences for Children and Adults, University of Modena and Reggio Emilia, Via Largo del Pozzo 71- 41124 Modena, Italy; b Division of Plastic Surgery, Department of General Surgery and Surgical Specialties. University of Modena and Reggio Emilia, Via Largo del Pozzo 71, 41124 Modena, Italy; c Internal Medicine and Critical Care Unit, Department of Integrated Medicine, Emergency Medicine and Medical Specialties, University of Modena and Reggio Emilia, Via Largo del Pozzo 71, 41124 Modena, Italy

**Keywords:** Pectus excavatum, Surgical debridement, Wound infection, VAC therapy, Modified Ravitch

## Abstract

**Introduction:**

Multiple surgical debridement sessions are mandatory before wound closure in cases of infection after a modified Ravitch procedure for pectus excavatum. Vacuum-assisted closure (VAC) is a well-established technical resource for treating complicated wounds; however, in cases of suspicion of bone infection, this approach is not enough to prevent bar removal.

**Presentation of the case:**

We present a case of surgical wound dehiscence with hardware exposure in a patient who had undergone chondrosternoplasty for pectus excavatum. Several sessions of debridement (three) and VAC were applied every time. The final result was achieved without the necessity to remove the hardware; however, to avoid the risk of infection, a bilateral pectoralis muscle flap mobilization was performed as the final step after the surgical wound revisions, although this approach is suggested to be used during the modified Ravitch procedure. This approach allows for a significant reduction in late complications and improves morphological outcomes.

**Discussion:**

In summary, the pectoralis muscle flap transposition is very useful not only for aesthetical results but also in combination with multiple surgical revisions for conservative management in case of wound infection during a modified Ravitch procedure. In our case, this technique was adopted after accurate care of the wound and before the final closure, which helps to maintain good vascularization and a very satisfying result.

**Conclusion:**

It is important to consider this approach during the modified Ravitch procedure, not only for better aesthetical results but also to prevent infections or wound dehiscence at the level of the bar.

## Highlights

• Vacuum-assisted closure is a well-established technical resource for treating complicated wounds.
• In cases of suspicion of bone infection, VAC therapy is not enough to prevent bar removal.
• Multiple surgical debridement sessions are mandatory before wound closure in cases of infection.
• We present a case of surgical wound dehiscence with hardware exposure.
• After VAC therapy and surgical debridement, the bilateral pectoralis muscle flap mobilization has been used.

## 1 Introduction

Pectus excavatum is a structural deformity of the anterior thoracic wall in which the sternum and rib cage are shaped abnormally. This produces a caved-in or sunken appearance of the chest. It can either be present at birth or develop after puberty. As described by many authors [[1], [2], [3]], metal supports for internal fixation to stabilize the sternum in the new corrected position are used at our institution. The modified Ravitch procedure is a very invasive technique, but it is still the best solution in cases of severe deformity of the sternum.

Infections of the wound are not very frequent; however, it is very dangerous to keep the bars inside in cases of wound infection due to the risk of severe complications, such as bone infection. In the scientific literature, the vacuum-assisted closure (VAC) procedure is a well-defined technique used in cases with suspicion of wound infection because the aspiration serves to keep the wound clean [4,5]. However, we believe that it is not sufficient to avoid infection, especially regarding the sternum. In this case report, the importance of performing surgical debridement multiple times in cases of wound infection after a modified Ravitch procedure for pectus excavatum has been emphasized because the surgeon decided not to remove the bars. VAC therapy is important to perform as soon as the clinical condition has been stabilized; however, it is not enough to prevent infection. We believe that pectoralis muscle flap transposition [[6], [7], [8], [9]] was the correct approach combined with multiple debridements and VAC therapy to treat this patient without the necessity to remove the bars.

The final results after one year were good in terms of both aesthetic skin closure and patient satisfaction. This work has been reported in line with the SCARE criteria [10].

## 2 Case presentation

**Fig. 1:**
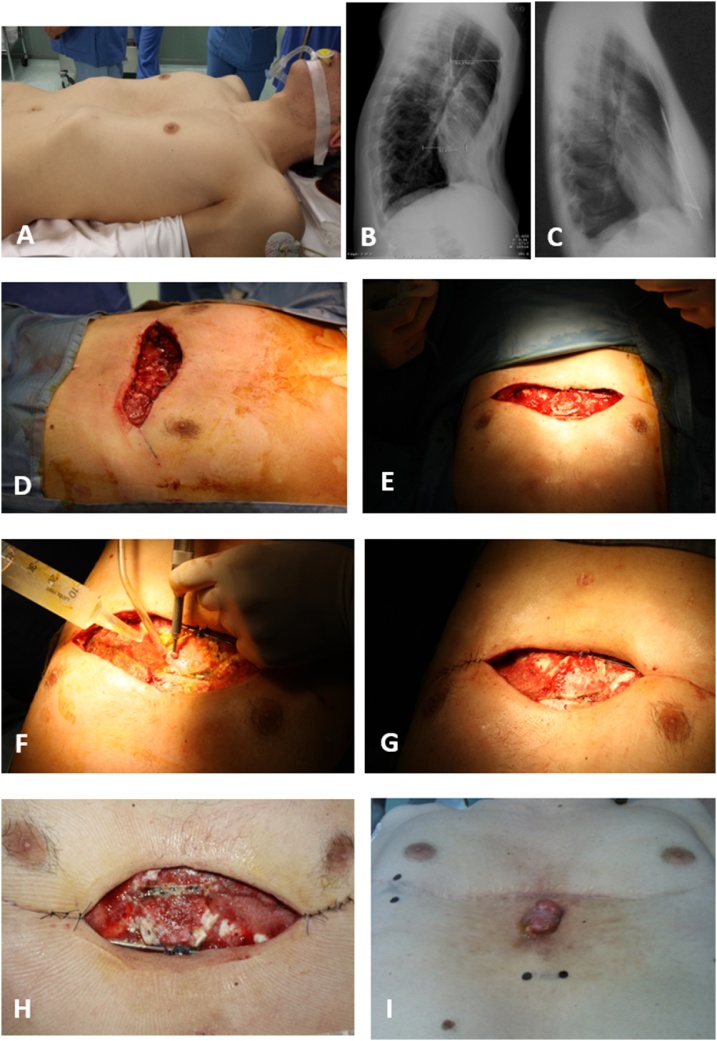
A. Patients before surgery. 1B. Pectus deformity is clearly visible in supine position as well as by chest x-ray. 1C. Chest x-ray after surgery and before bars removal, showing a satisfactory result with no hardware exposure. 1D. The wound before the first-time surgical debridement. 1E. The wound appears red and full of serum. 1F-G. The debridement and medication during the second surgical debridement. 1H. Third time surgical debridement. 1I. The wound appeared less red and without serum. At this time the skin was closed after 10 days of VAC therapy.

A 24-year-old male underwent a modified Ravitch procedure for pectus excavatum for a persistent referred tachycardia and dyspnea on exertion (Fig. 1A, B). Preoperative chest CT showed a severe pectus excavatum with Haller index equal to 3.44. For the first time, the pectoralis muscle flap mobilization adapted to the modified Ravitch technique for pectus excavatum reconstruction has been shown with no complications. After being discharged on the eighth postoperative day, the patient returned to our hospital due to excessive serum exiting from the surgical wound. A clinical examination showed that the hardware was exposed. Microbiological samples from the wound were negative, and the patient was apyretic, with no general symptoms. Therapy with a broad-spectrum antibiotic was introduced. In accordance with the plastic surgeon, the patient underwent surgical debridement multiple times (three times) to prevent infection of the bone (Fig. 1D–H). VAC therapy was performed every time after debridement. The patient was then referred to the Plastic Surgery Unit. The final closure of the wound was performed 10 days after the last debridement.

**Fig. 2:**
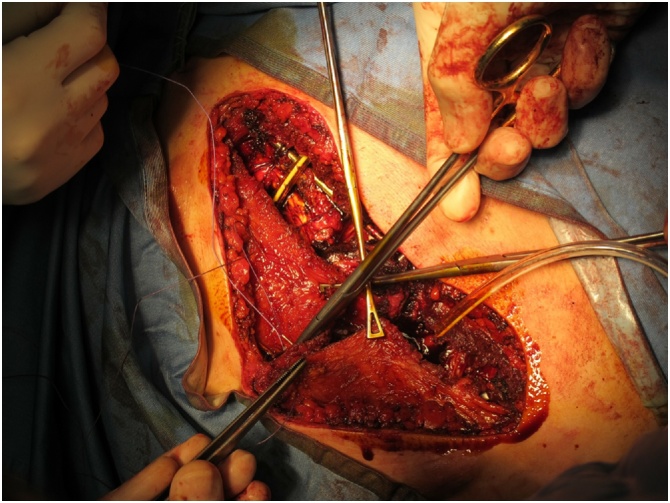
Pectoralis muscle mobilization and suture.

**Fig. 3:**
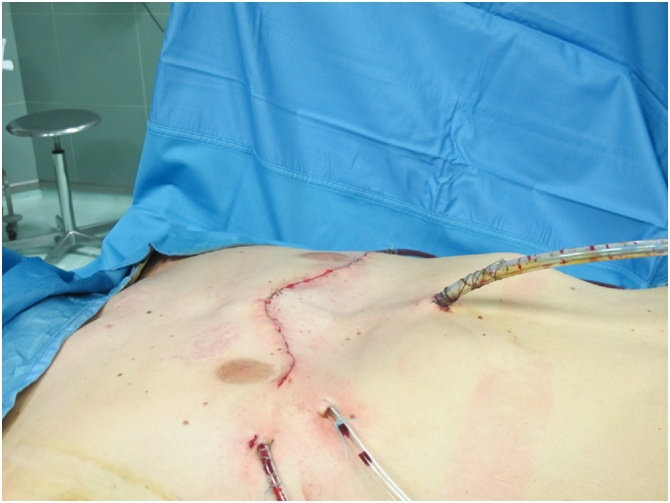
Final closure after pectoralis muscle mobilization.

At this time, a pectoralis muscle flap transposition was performed to reduce the risk of infection, protecting the bar with the muscle layer. Both pectoralis muscles were carefully dissected on a superficial prefascial plane from the overlying skin and subcutaneous layer. When proceeding cranially, care was taken not to devascularize the skin flap. The pectoralis muscles were elevated, and the thoracoacromial pedicle was identified and preserved. Muscles were then mobilized as needed to reach a comfortable lateral-to-medial rotation/transposition. Once the flaps had been fully mobilized, hemostasis was accurately controlled, and the two flaps were sutured to one another medially with PDS sutures (Fig. 2). With this, full muscular coverage of the osteotomized sternum and ribs was obtained. The hardware was also almost fully protected by this maneuver. Two submuscular drains were inserted, and the muscles were sutured inferiorly to the deep fascia or to the rectus muscle fascia to obtain complete muscular coverage of all the underlying elements. Final closure was thus obtained with skin sutures in a double layer (Fig. 3).

The patient was discharged in good condition after 10 days with no further complications. At one month after surgery, an outgrowth from the wound appeared at the level of the sternum with the release of clear serum, which was negative on microbiological examination and required removal under general anesthesia after patient hospitalization (Fig. 1I). The one-year follow-up examination showed no more complications and total resolution of the surgical scars. Chest X-ray showed radiological findings indicating complete stability, with no hardware exposure (Fig. 1C).

## 3 Discussion and conclusion

Sternochondroplasty is a standard procedure for the correction of pectus excavatum deformity [[1], [2], [3]]. Although nowadays Nuss minimal invasive procedure is considered the standard procedure in many centers, the scientific literature underlines the value of the modified Ravitch procedure to reduce the risk of complications after surgery [11,12]. In fact, in 2016 Kanagaratnam et al. in a systematic review and meta-analysis suggested no differences between Nuss and Ravitch procedures for pediatric patients, although in adults the Ravitch procedure resulted in fewer complications [11,12]. For this reason, the open approach and the pectoralis muscle transposition have been chosen.

Wound complications in the presence of hardware may be devastating functionally, aesthetically, and psychologically given the high expectations of the patient. Salvage procedures are indicated only in cases of negative serum microbiological cultures, no fever, and no clinical complications. If the patient becomes pyretic with a positive result for bacterial infection at the level of the surgical wound, the bars must be removed immediately to prevent severe infection. In our case, we opted for a salvage approach with an accurate clinical assessment of the patient associated with multiple debridement sessions in the operative room under general anesthesia.

Performing debridement multiple times is a very important solution and VAC therapy may improve the possibility of saving the hardware, thus preserving the sternum in the corrected position [[4], [5], [6]]. However, this approach may not be enough in the case of dehiscence of the wound. To avoid infection and removal of the bar, we showed in practice for the first time the utility of using the pectoralis flap mobilization, which guarantees a better coverage of the bars associated with a good aesthetical result, especially in the case of thin patients [7].

In fact, this approach allows for a significant improvement of the final outcome by providing a well-vascularized layer protecting hardware, supporting bone and cartilage healing, and improving soft tissue thickness. Furthermore but less importantly, vascularized muscle represents an ideal site for adipose cell graft removal in case of need. Further studies will need to be undertaken to show the real effect and benefits of this new surgical approach.

## Sources of funding

No funding.

## Author contribution

AB and BA wrote the case report. GDS, LB, AS, CR and UM revised the case report.

## Research studies

Ethical Board approval is not required for case reports in our Center.

## Declarations

Availability of supporting data: yes.

## Ethics approval and consent to participate

Ethical Board approval is not required for case reports in our Center.

## Consent for publication

Consent for publication: written informed consent was obtained from the patient for the publication of this case report and for any images. A copy of the written consent is available for review by the Editor-in-Chief of this journal on request.

## Provenance and peer review

Not commissioned, externally peer-reviewed

## Declaration of Competing Interest

The Authors have no financial and personal relationships to disclose.
